# Structural Characterization and Anti-Inflammatory Activity of a Bioactive Polysaccharide from *Cistanche deserticola*

**DOI:** 10.3390/foods15152735

**Published:** 2026-08-04

**Authors:** Baotang Zhao, Faqin Tao, Yongpei Xiao, Guofeng Li, Mingze Li, Yulong Huang

**Affiliations:** 1 College of Food Science and Engineering, Gansu Agricultural University, Lanzhou 730070, China; 2 College of Life Science, Northwest Normal University, Lanzhou 730070, China; faqintao@126.com; 3 Institute of Agricultural Products Storage and Processing, Gansu Academy of Agricultural Sciences, Lanzhou 730070, China

**Keywords:** polysaccharides, structural characterization, anti-inflammatory, metabolomics

## Abstract

A bioactive polysaccharide (CDP-D1) was isolated from the bulbs of *Cistanche deserticola* and purified by DEAE anion-exchange chromatography, yielding a purity of 95.5% and a recovery rate of 33.1%. It exhibits an irregular amorphous structure, with a porous and loose surface, and displays distinct shear-thinning behavior. Its molecular weight (MW) is 142.52 kDa, and its polydispersity index (Mw/Mn) is 1.77, confirming its heterogeneity. Gas chromatography–mass spectrometry (GC-MS) and nuclear magnetic resonance (NMR) analysis identified it as a heteropolysaccharide. Functional studies indicate that CDP-D1 mediates a protective effect against lipopolysaccharide (LPS)-induced inflammation in RAW264.7 macrophages. CDP-D1 restored LPS-induced decreases in cell viability to near-normal levels and suppressed pro-inflammatory signaling by downregulating TLR4 mRNA, key cytokines (IL-1, IL-6, TNF-α), and their cascade amplification. Metabolomic analysis (PLS-DA) validated the reliability of the model and identified 420 metabolites. LPS induced metabolic suppression, whereas CDP-D1 reversed this trend. Key pathways regulated by CDP-D1 include glutathione metabolism and amino acid/nucleoside/purine metabolism, partially restoring metabolic homeostasis. These findings provide preliminary evidence that CDP-D1 modulates inflammatory responses in LPS-stimulated macrophages, potentially through regulation of oxidative stress and metabolic reprogramming, and warrant further investigation into its molecular mechanism of action.

## 1. Introduction

*Cistanche deserticola*, commonly known as “desert ginseng,” is a plant of the genus Cistanche (family Orobanchaceae), recognized as a medicinal and edible dual-purpose species. It is primarily distributed in China (Xinjiang, Inner Mongolia, Gansu), Mongolia, Iran, and other regions. Cistanche was first documented in the *Shennong Bencao Jing* (*Shennong’s Classic of Materia Medica*), and due to its remarkable medicinal properties and tonic effects, it has long been utilized as a traditional Chinese herb and health food [1,2,3,4]. Currently, it is widely employed as a raw material in the development of functional foods and health products. Since ancient times, Cistanche has been used to treat kidney-deficiency-related disorders, including sexual dysfunction, female infertility, deficiency of essence and blood, aching in the lower back and legs, dryness of the bowels, and constipation. It has been included in *Catalogue of Drugs and Foods Homologous in China*. Modern pharmacological studies have shown that Cistanche polysaccharides exhibit diverse biological activities, such as anti-aging, antidepressant, hepatoprotective, blood glucose-lowering, lipid-lowering, antitumor, anti-inflammatory, anti-osteoporotic, and anti-fatigue activities [5,6,7,8].

To date, a total of 16 homogeneous polysaccharides have been isolated and structurally characterized from *Cistanche*. According to the presence or absence of uronic acid moieties, these polysaccharides can be classified into neutral polysaccharides and acidic polysaccharides, with acidic polysaccharides constituting the predominant homogeneous polysaccharide components of *Cistanche*. Wu et al. isolated a polysaccharide-designated ACDP-2 from the aqueous extract of *Cistanche*, which is a highly branched arabinogalactan polymer composed of (1→4)-linked D-galactose (D-Gal) and D-glucose (D-Glc) residues, featuring a branching point at the C-6 position. The branched side chains consist of amino-substituted, 1,5-linked, and 1,3,5-linked arabinofuranosyl (Araf) residues, and this polysaccharide exhibits dose-dependent induction of cell proliferation [9]. In addition, the same team identified a neutral polysaccharide, CDP-4, which is a linear dextran with an average molecular weight (Mw) of 14 kDa. CDP-4 is primarily composed of (1→4)-linked glucopyranosyl (Glcp) and (1→6)-linked Glcp residues in a molar ratio of 1:3. The main chain of the novel mannan polysaccharide CDP-6 consists of (1→6)-linked glucose and mannose residues, with terminal (1→6)-glucose and (1→3,6)-mannose residues attached at the O-3 position of mannose (Man) units.

Research has reported that natural products derived from traditional Chinese medicine can exert both anti-inflammatory effects and block LPS-induced metabolic reprogramming through the NF-κB, MAPK, and STAT3 pathways [10]. Natural products found in food and medicinal plants may also restore metabolic homeostasis by regulating glutathione metabolism and amino acid/nucleoside/purine metabolic pathways [11,12,13]. Unlike studies that focus on a single signaling pathway, this study links the physicochemical properties of *Cistanche deserticola* polysaccharides—such as their heterogeneous polydisperse structure and shear-thinning rheological behavior—to their unique functions in regulating immune metabolism, thereby demonstrating anti-inflammatory effects at the metabolism–immunity interface. Regarding the mechanisms by which natural products inhibit TLR4 activation and modulate TLR4-mediated inflammatory responses, the relationship between the structure and activity of natural bioactive compounds, such as polysaccharides, remains to be further investigated; exploring the interaction patterns of the TLR4/MD-2 complex will be an important direction for future research [14,15,16,17]. These findings provide preliminary evidence that CDP-D1, a pectin-like heteropolysaccharide from *Cistanche deserticola*, attenuates inflammatory gene expression and partially normalizes metabolic dysregulation in LPS-stimulated RAW264.7 macrophages. While these results warrant further investigation, they represent an early step toward understanding the biological potential of *Cistanche deserticola* polysaccharides. Translation of CDP-D1 into practical applications for inflammatory disease management will require extensive follow-up studies, including in vivo efficacy validation, bioavailability assessment, and safety evaluation. While our previous work provided a preliminary overview of the extraction and general bioactivities of polysaccharides from *Cistanche deserticola* [18], the specific structural attributes and anti-inflammatory mechanisms of the individual fractions remained largely unexplored. Notably, the first-eluted neutral fraction, designated CDP-D1, was not extensively characterized. Therefore, this study focuses exclusively on the in-depth structural elucidation of CDP-D1 using methylated analysis and NMR spectroscopy, coupled with an evaluation of its anti-inflammatory effects on LPS-induced RAW264.7 cells, aiming to establish a proposed relationship between the structural characteristics and biological activity of CDP-D1. Additionally, the study evaluates its effects on cellular activity and performs untargeted metabolomics analysis to delineate metabolic profile alterations in LPS-induced RAW264.7 cells.

## 2. Materials and Methods

### 2.1. Materials and Reagents

*Cistanche deserticola* polysaccharide (CDP-D1) was prepared by the previously reported method in this laboratory [18,19].

Lipopolysaccharide (LPS) (Sigma-Aldrich, St. Louis, MO, USA), RAW264.7 (Chinese Academy of Sciences Cell Bank, Shanghai, China), fetal bovine serum, culture medium (DMEM), double antibiotic (streptomycin and penicillin) and trypsin (Gibco, Thermo Fisher Scientific, Waltham, MA, USA), TNF-α, IL-6 and IL-1 kits, RNA kits, fluorescence quantification kits (Solarbio, Beijing, China; Shanghai branch, Shanghai, China), RIPA cell lysate and PMSF protease inhibitor (Beyotime, Shanghai, China), primary antibody dilution (Shanghai Biyuntian Biotechnology Co., Ltd., Shanghai, China), Enzyme Labeler, microplate reader (Bio-Rad, Hercules, CA, USA); thermal cycler (T100™, Bio-Rad, Hercules, CA, USA), Quantitative PCR (Thermo Fisher Scientific, Waltham, MA, USA), Nitrogen Purge (Reacti-Vap, Thermo Fisher Scientific, Waltham, MA, USA), Ion Chromatography (Dionex ICS-5000+, Thermo Fisher Scientific, Waltham, MA, USA), Vortex Mixer (XH-T, Changzhou Langyue Instrument Co., Ltd., Changzhou, Jiangsu, China).

### 2.2. Experimental Methods

#### 2.2.1. Preparation of Polysaccharide

A crude polysaccharide extract was prepared, subjected to proteolytic digestion, and purified using the Savage method to remove proteins. Petroleum ether was added for defatting, and microporous resin AB-8 was used for decolorization. A 3000 Da dialysis membrane was used to remove small-molecule substances. Finally, following the method described by Zhao et al., the final product was freeze-dried, resuspended, and further purified using DEAE-cellulose; the first fraction was selected and named CDP-D1 [18].

#### 2.2.2. Infrared Spectral Scanning

The CDP-D1 and KBr (infrared-grade) were accurately weighed and thoroughly mixed in an agate mortar. The mixture was pressed into a 1 mm thick transparent sheet using a hydraulic press at 10 MPa. The sheet thickness was measured as described.

#### 2.2.3. Molecular Weight Determination

Molecular weight determination was performed using a gel chromatography–differential refractive index–multiphase laser light scattering (GC-DR-MPLS) system. This method was used to determine key parameters of the polysaccharide CDP-D1, such as molecular weight (Mw), z-average molecular weight (Mz), number-average molecular weight (Mn), and polydispersity index (Mw/Mn), to ensure standardization of molecular characterization [18,20,21].

#### 2.2.4. Determination of Monosaccharide Composition

The monosaccharide composition and molar ratios of CDP-D1 were primarily determined using high-performance liquid chromatography (HPAEC), a method that involves trifluoroacetic acid (TFA) hydrolysis, purification, and ion chromatography (IC) analysis [18,20,21].

#### 2.2.5. Methylation Analysis

Methylation analysis was primarily performed using GC-MS, with the temperature range set at 140–230 °C, a heating rate of 3 °C/min, and a mass scan range of 50–350 *m*/*z* [18].

#### 2.2.6. Differential Scanning Thermal Analysis

The method of Reihaneh et al. was adopted [22]. Approximately 5 mg of CDP-D1 was evenly spread on the bottom of an aluminum plate, which was then sealed. The temperature was increased from 25 to 200 °C at a rate of 10 °C/min. The measured data were processed to obtain the DSC curve.

#### 2.2.7. Thermogravimetric Analysis

The method of Cao et al. was adopted [18,23]. The thermogravimetric analysis of polysaccharides was performed using a TGA55 thermal analyzer. CDP-D1 weighing 5–7 mg was placed in a crucible, with nitrogen gas as the carrier to prevent thermal oxidation. The heating temperature ranged from 25 °C to 600 °C, with a heating rate of 20 °C/min.

#### 2.2.8. Analysis of Rheological Properties

Based on Wang’s methodology with modifications [24], the rheological properties of CDP-D1 solution were investigated using an HR-1 rheometer. During shear rate and frequency scans, the temperature was maintained at 25 °C, while temperature scans were conducted across a range from 25 to 100 °C [24,25].

#### 2.2.9. Morphological Analysis

Morphology analysis of the CDP-D1 was performed according to the method previously reported. The magnification range was set from ×500 to ×10,000, and high-resolution images were collected to analyze the surface microstructure of CDP-D1 [18].

#### 2.2.10. NMR Analysis

The primary structure of the polysaccharides was determined using a Bruker Avance III HD 500 MHz nuclear magnetic resonance (NMR) spectrometer for multidimensional NMR analysis. One-dimensional ^1^H/^13^C spectra and two-dimensional intranuclear/exonuclear correlation spectra (COSY, HSQC, HMBC) were acquired to elucidate bonding patterns and glycosidic bond configurations, with NOESY spectra used to assist in determining local molecular conformations [18,21].

#### 2.2.11. Cell Viability Assay

The CCK-8 assay is primarily used to assess cell viability. RAW264.7 cells were seeded in 96-well or 6-well plates and cultured in DMEM/F-12 medium containing 10% fetal bovine serum (FBS) at 37 °C in a 5% CO_2_ incubator. After 24 h of incubation, the cells were divided into four groups: (1) a normal control (NC) group, receiving no treatment; (2) a model (LPS) group, treated with Escherichia coli O55:B5 lipopolysaccharide (LPS, 10 μg/mL, Merck, Darmstadt, Germany) for 12 h; (3) a CDP-D1 treatment group, pretreated with CDP-D1 (125 μg/mL) for 12 h prior to LPS stimulation (10 μg/mL, 12 h); and (4) a blank control group. Cell viability was assessed using the Cell Counting Kit-8 (CCK-8, Dojindo Laboratories, Kumamoto, Japan). After treatment, 10 μL of CCK-8 reagent was added to each well, and the plates were incubated for an additional 2 h. The optical density (OD) was measured at 450 nm using a microplate reader (Bio-Rad Instruments, Hercules, CA, USA). Cell survival rates were calculated based on the OD values. For the collection of supernatants and cells, after the 12 h co-incubation period, the culture media were harvested for ELISA analysis, and the cells were collected for RNA extraction and qPCR analysis. All experiments were performed with at least three independent biological replicates [18,26,27].

#### 2.2.12. Assessment of Inflammatory Cytokine Expression

The effects of the polysaccharide CDP-D1 on the mRNA expression levels of inflammatory cytokines (IL-6, IL-1β, TNF-α, and TLR4) in LPS-induced RAW264.7 macrophages were determined using qPCR. Total RNA was extracted from RAW264.7 cells using TRIzol reagent (TRIzol™, Invitrogen, Thermo Fisher Scientific, Carlsbad, CA, USA) according to the manufacturer’s protocol. cDNA was synthesized using a PrimeScript RT Reagent Kit (PrimeScript™, Takara Bio Inc., Otsu, Shiga, Japan). Quantitative real-time PCR (qPCR) was performed using SYBR Premix Ex Taq (TaKaRa) on a CFX Connect Real-Time PCR System (Bio-Rad). The primer sequences used for amplification are listed in Table 1. The cycling conditions were as follows: 95 °C for 30 s, followed by 40 cycles of 95 °C for 5 s and 60 °C for 30 s. The relative expression levels of target genes (IL-6, IL-1β, TNF-α, and TLR4) were calculated using the 2^−ΔΔCt^ method and normalized to the internal reference gene *β*-actin [18,21,28,29].

#### 2.2.13. Untargeted Metabolomics Analysis

Untargeted metabolomics analysis was performed to compare metabolic profiles among control (CK), LPS-induced inflammation (LPS), and CDP-D1 polysaccharide-treated (CDP-D1) RAW264.7 cells. Raw mass spectrometry (MS) data were acquired using a Q-Exactive instrument controlled by Xcalibur 4.1 software, and processed via Progenesis QI for peak extraction, alignment, and normalization, with quantified data exported to Excel format. Data analysis was conducted using R packages, involving orthogonal partial least-squares discriminant analysis (OPLS-DA) to construct classification models for intergroup comparison (CK vs. LPS vs. CDP). Model robustness and overfitting risk were evaluated via 7-fold cross-validation and response permutation testing. Variable importance in the projection (VIP) values was calculated to identify metabolites contributing to group discrimination, with VIP > 1 selected as potential differential metabolites.

#### 2.2.14. Statistical Analyses

Significant differences between control and LPS-induced RAW264.7 in the normal state were determined by origin 2021 software.

## 3. Results and Analysis

### 3.1. Separation and Purification of CDP-D1

Following the established protocol, crude CDP was fractionated using DEAE-cellulose chromatography, yielding four fractions (CDP-D1 to CDP-D4). While our previous study focused on the mixed properties of these fractions, this work specifically targets the first eluted fraction, CDP-D1 [1]. Preliminary analysis indicated that CDP-D1 possessed the highest homogeneity and unique structural features; thus, it was selected for comprehensive characterization and bioactivity assays in the present study. When eluted with pure water, the first component (CDP-D1) exhibited a purity of 95.5% and a yield of 33.1% [18].

### 3.2. FT-IR

**Figure 1:**
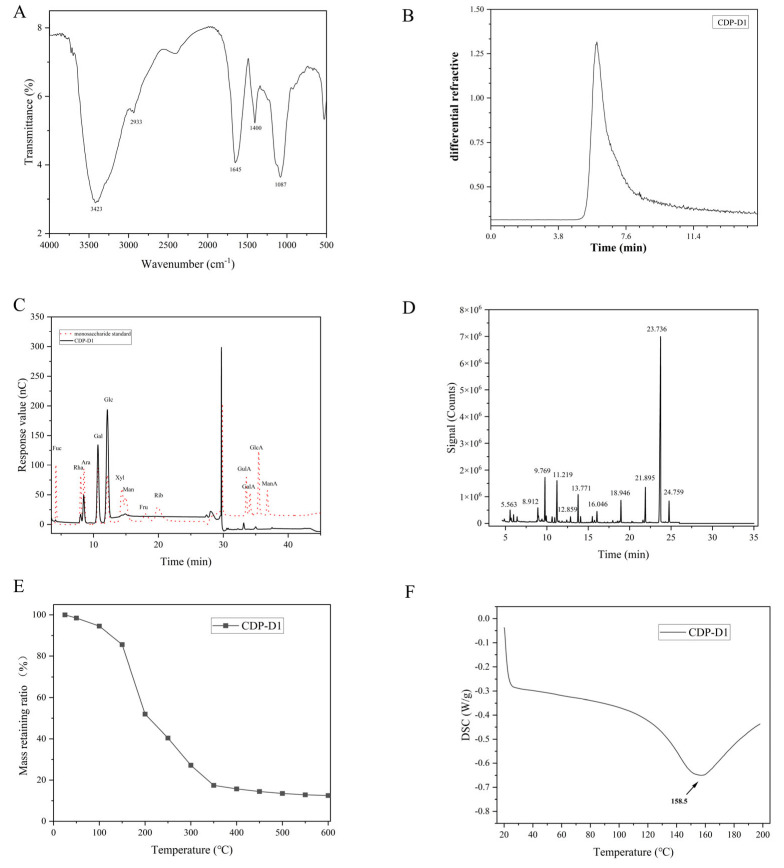
FT-IR spectrum scanning (**A**), HPGFC chromatogram (**B**), monosaccharide composition (**C**), total ion chromatogram (**D**), TG curve (**E**) and DSC curve (**F**) of CDP-D1.

As shown in Figure 1A, CDP-D1 exhibits absorption peaks typical of polysaccharides in the range of 3600–500 cm^−1^. The absorption peaks at 3423 cm^−1^ correspond to O–H vibrational peaks, while those at 2933 cm^−1^ correspond to C–H stretching vibrations. The absorption peaks at 1645 cm^−1^ can be attributed to the C=O groups in carboxyl or ester groups, indicating the presence of uronic acid in CDP-D1 [30]. The characteristic absorption peaks at 1087 cm^−1^ correspond to the overlapping vibrations of the C-O-C and C-O-H bonds in the glycosidic bond [31].

### 3.3. Molecular Weight

**Figure 2:**
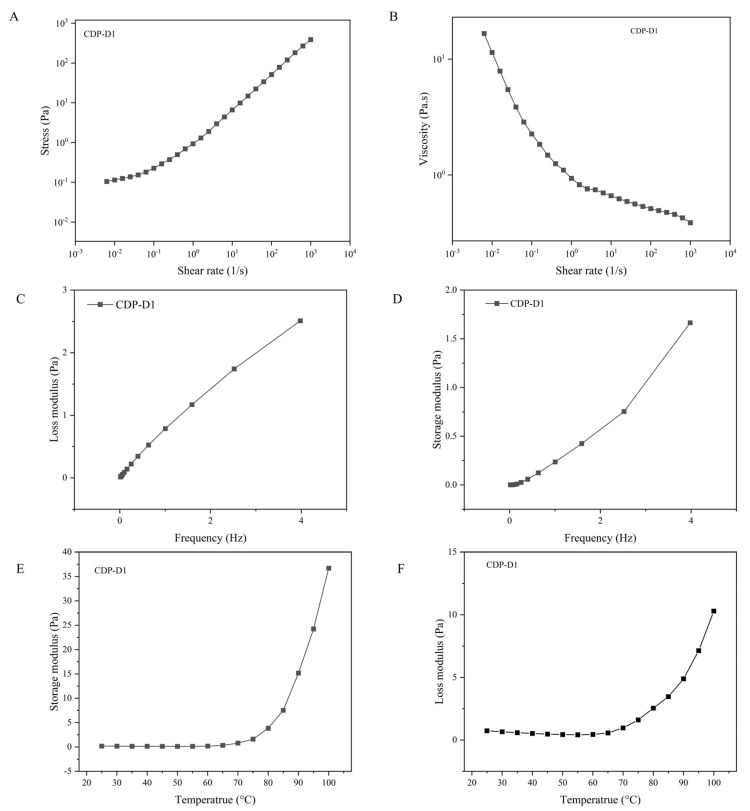
Rheological curves of CDP-D1. (**A**) Shear rate–shear stress; (**B**) shear rate–viscosity; (**C**) frequency-loss modulus (G″); (**D**) frequency-storage modulus (G′); (**E**) temperature-storage modulus (G′); (**F**) temperature-loss modulus (G″).

As shown in Figure 2B, the weight-average molecular weight (Mw), z-average molecular weight (Mz), and number-average molecular weight (Mn) of CDP-D1 are 142.52 kDa, 158.22 kDa, and 178.42 kDa, respectively. The polydispersity indices (Mw/Mn) of CDP-D1 were 0.80, indicating that CDP-D1 has narrow molecular weight distributions and relatively uniform polysaccharides. This result is inconsistent with Qiu’s report, in which Qiu et al. indicated that the polydispersity index (Mw/Mn) of CDPs was 6.34, suggesting a narrow molecular weight distribution of the polysaccharides [32]. At the same time, Qiu et al.’s molecular weight measurement results showed that the Mw, Mz, and Mn of CDP were (4.11 ± 0.02) × 10^5^, (2.45 ± 0.03) × 10^6^, and (6.48 ± 0.04) × 10^4^, respectively. Compared to small-molecule compounds, polysaccharides do not have a fixed molecular weight; they are mixtures containing molecules of varying molecular weights. Because the molecular weight of polysaccharides is not fixed, it may be influenced by homologs.

### 3.4. Monosaccharide Composition and Molar Ratios

As shown in Figure 1C, CDP-D1 consists primarily of Ara, Rha, Gal, Glc, and Gal-UA, with molar ratios of 8.86:3.42:27.23:57.07:2.64. This result clearly indicates that CDP-D1 consists of heterogeneous polysaccharides, with Gal and Glc being the major components of CDP-D1. This composition is inconsistent with the findings of Qiu and Li. Qiu et al. identified six monosaccharides—Rha, Ara, Gal, Glc, Man, and Glc-UA—in the CDPs, with a molar ratio of 14.4:0.6:5.6:99.7:4.3:0.3. Meanwhile, Li et al. found six monosaccharides in CDPs: Glc, Man, Rha, Gal, GlcA, and Ara, with a molar ratio of 33.17:2.42:10.97:16.39:8.05:28.98 [32,33]. The significant differences in monosaccharide composition and molar ratios between this study and previous ones can be attributed to three main factors. First, unlike previous studies that analyzed crude extracts, CDP-D1 is a specific, purified neutral fraction obtained via DEAE–cellulose chromatography, which selectively enriches for specific structural classes of polysaccharides. Second, the source and variety of Cistanche deserticola used in this study may differ from those in previous reports, as environmental factors and harvest time significantly influence polysaccharide biosynthesis. Third, variations in extraction protocols and analytical methodologies can lead to quantitative discrepancies. Therefore, the unique profile of CDP-D1 underscores its identity as a refined bioactive entity rather than a reflection of contradictory data.

### 3.5. Glycosidic Linkage Analysis by Methylation-GC-MS

GC-MS analysis was used to examine the methylation of CDP-D1; the results for sugar residues and glycosidic bonds are shown in Figure 1D and Table 1. CDP-D1 contains 3,4-Gal(p), 2,4-Rha(p), 1, 6-Glc(p), 3CDP-Gal(p), 1,4-Gal(p)-A, t-Gal(p), 5-Ara(f), 1,4-Glc(p), 6-Glc(p), and 3,6-Gal(p), with the major components being 1,6-Glc(p), 1,4-Glc(p), and 3-Gal(p).

**Table 1:** Methylation analysis of CDP-D1.

Type of Linkage	Methylated Sugar	Mass (M_W_)	Percentages (%)
3,4-Gal(p)	1,3,4,5-tetra-O-acetyl-2,6-di-O-methyl galactitol	333	6.54
2,4-Rha(p)	1,2,4,5-tetra-O-acetyl-6-deoxy-3-O-methyl rhamnitol	354	4.35
1,6-Glc(p)	4,5-di-O-acetyl-2,3di-O-methyl glucitol	341	34.11
3-Gal(p)	1,3,5-tri-O-acetyl-2,4,6-tri-O-methyl galactitol	344	13.08
1,4-Gal(p)-A	1,4,5-tri-O-acetyl-2,3,6-tri-O-methyl galactitol	323	6.51
t-Gal(p)	1,5-di-O-acetyl-2,3,4,6-tetra-O-methyl galactitol	369	2.70
5-Ara(f)	1,4,5-tri-O-acetyl-2,3-di-O-methyl arabinitol	335	2.30
1,4-Glc(p)	1,4,5-tri-O-acetyl-2,3,6-tri-O-methyl glucitol	341	24.23
6-Glc(p)	1,4,5-tri-O-acetyl-2,3,6-tri-O-methyl glucitol	365	2.73
3,6-Gal(p)	1,3,5,6-tetra-O-acetyl-2,4-di-O-methyl galactitol	345	3.22

### 3.6. Thermal Performance Analysis

Differential scanning calorimetry (DSC) analysis has the advantages of a wide range of applications and easy and time-saving operation, and is an effective means of study for measuring the change in the power difference of a sample in relation to temperature under programmed temperature control. The DSC curve of CDP-D1 is shown in Figure 1E. It can be seen that in the temperature interval of 25–100 °C, the DSC curves show an obvious decreasing trend with a turning point, which is mainly due to the volatilization of volatile substances such as CDP-D1. In the temperature interval of 100–200 °C, the peak heat absorption of CDP-D1 was 158.5 °C.

The thermogravimetric (TG) method of analysis is the determination of the change in mass of a sample with temperature when the temperature is controlled by a programmer. Figure 1F shows the TG curve of CDP. It can be seen that the weight loss process of CDP-D1 can be roughly divided into the following three stages. The initial weight loss temperature is about 25~150 °C, and the weight loss rate of CDP-D1 is about 15.2%, and the weight loss in this stage is mainly caused by the evaporation of water and the volatile components volatilization [34]. The second stage, which is the most dominant weight loss region, occurs in the temperature interval of 100~350 °C, where the weight loss rates are all up to 75% or more, and the main reason for the substantial weight loss is the thermal decomposition of CDP-D1. The final stage is in the temperature interval of 350~600 °C, where CDP-D1 will continue to undergo thermal decomposition.

### 3.7. Rheological Analysis

Energy storage modulus (G′) refers to the positive correlation between the real part of the complex modulus and its maximum elastic energy in materials with elastic-viscous properties in each stress or each strain cycle, which is generally used to reflect the resilience index of the material after deformation; loss modulus (G″) refers to the positive correlation between the imaginary part of the complex modulus and its damping loss factor in materials with elastic-viscous properties, which is generally used to reflect the material’s dissipation of the deformation energy. It is generally used to reflect the magnitude of the dissipated deformation energy of the material, which can reflect the viscosity of the material [35,36]. The tangent of the loss angle is usually expressed using tan δ, which represents the ratio of the energy lost by the medium to its stored energy per cycle [37].

As can be seen from Figure 2A, the viscosities of CDP-D1 are highly dependent on the shear rate, and the apparent viscosity of the system exhibits a significant power-law dependence on the shear stress under the condition of continuously increasing shear rate, and their nonlinear rheological behavior is in line with the typical characteristics of shear-thinning fluids, indicating that the molecular chain entanglement network undergoes a dynamic dissociation along with the shear action. This is due to the continuous shear behavior, which leads to the rearrangement between the CDP-D1 molecules, resulting in the phenomenon of increased fluidity and decreased viscosity. When the frequency is low, the CDP solution viscosity increases with increasing concentration. However, after a shear rate of 100 s^−1^, the apparent viscosities of CDP-D1 gradually converge to the same, which is attributed to the fact that the orientation between the molecules gradually converges under increasing shear rate, and the viscosities will then gradually level off.

A non-linear regression analysis of the viscosity-shear rate relationship in the steady-state shear test was carried out using the Cross eigenequation, and the results of the fitted parameters are summarized in Table 2. The equations are as follows:

$$\frac{\eta -\eta_{\infty }}{\eta_{0}-\eta_{\infty }}=\frac{1}{{\left(1+\tau c_{r\dot{\gamma }}\right)}^{n}}$$

In the equation: $\eta_{0}$ denotes zero shear viscosity, Pa.s; $\eta_{\infty }$ denotes infinite shear viscosity, Pa.s; $\tau c_{r}$ denotes the consistency coefficient; $\dot{\gamma }$ denotes the shear rate, s^−1^; and n denotes the Cross index.

As can be seen from Figure 2B and Table 3, for CDP-D1 solutions, the shear stress has a strong dependence on the shear rate, and there is an approximately linear growth trend between the shear stress and the shear rate. The present experimental observation shows that during the time-dependent increase in shear rate, the fluid motion rate of CDP-D1 solution is significantly accelerated, and this kinetic response directly leads to the synchronous enhancement of the shear stress generated within the fluid, and the increase is significantly positively correlated with the magnitude of the change in shear rate. Between 10^−3^ s^−1^ and 10^−1^ s^−1^, the apparent viscosity of CDP-D1 decreased by approximately 40%, reflecting the onset of shear-induced disentanglement of the polysaccharide chains. However, after 10^−1^ s^−1^, CDP-D1 gradually converged.

The Herschel–Bulkley model was fitted to the rheological data from the steady-state shear experiments by nonlinear regression analysis, and the fitted parameters obtained are shown in Table 3 equation

$$\sigma =\sigma_{y}{+K}_{HB}{\dot{\gamma }}^{n}$$

where σ denotes shear stress (Pa); $\sigma_{y}$ denotes yield stress (Pa); $K_{HB}$ denotes HB consistency coefficient, (Pa·s); $\dot{\gamma }$ denotes shear rate (s^−1^); n denotes the index of rheological properties.

The model fitting parameters in Table 3 show that R^2^ are all above 0.999, indicating a good fit of the Herschel–Bulkley model: n is all between 0 and 1, which corresponds to shear thinning, and the CDP-D1 solutions are pseudoplastic fluids.

The variation in G′ versus G″ for frequency scans of CDP-D1 solutions is shown in Figure 2C,D. In the frequency range from 1 to 4 Hz, the G′ and G′ of CDP-D1 solutions overlapped slightly at the beginning and gradually began to differ as the frequency increased.

As shown in Figure 2E,F, G′ and G″ of 1% *w*/*v* CDP-D1 solutions exhibited identical trends during temperature sweeps from 25 to 100 °C. Both moduli remained essentially invariant between 25 and 65 °C, then displayed a modest, progressive increase at higher temperatures. Crucially, the absolute values of G′ and G″ remained below 10 Pa even at 100 °C.

The observed thermal stiffening may reflect enhanced transient intermolecular interactions, such as hydrophobic associations or thermally induced conformational ordering of the polysaccharide chains. However, we emphasize that these moduli are 1–2 orders of magnitude lower than the typical threshold for percolated gel networks (>100 Pa), confirming that CDP-D1 does not form a permanent cross-linked structure under the tested conditions. The temperature-dependent increase therefore represents reversible, short-lived associative interactions rather than the formation of a denser network.

### 3.8. SEM of CDP-D1

**Figure 3:**
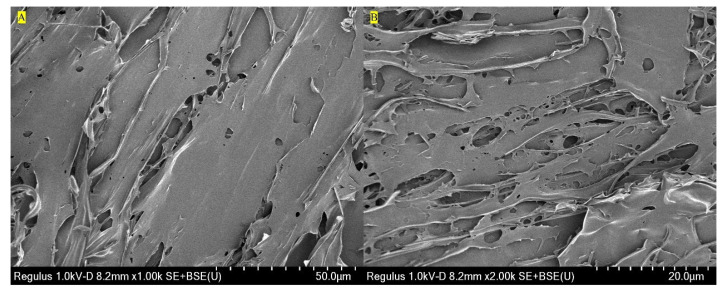
SEM image of CDP-D1 at 50 μm scale bar (**A**) and 20 μm scale bar (**B**).

As shown in Figure 3, scanning electron microscope images reveal the surface morphology of the polysaccharide. CDP-D1 consists primarily of irregular lamellae. This structure of CDP is related to its characteristics as a xerophyte. It is precisely the presence of this three-dimensional network-gel structure that enhances the interactions between hydroxyl groups and water molecules, thereby increasing its water absorption and water-holding capacity. Consequently, it is able to adapt to arid and decertified environments and exhibits drought tolerance. According to the research by Guo et al., morphological differences in polysaccharides are attributed to variations in their structural composition [38].

### 3.9. Structure Analysis of CDP-D1

**Figure 4:**
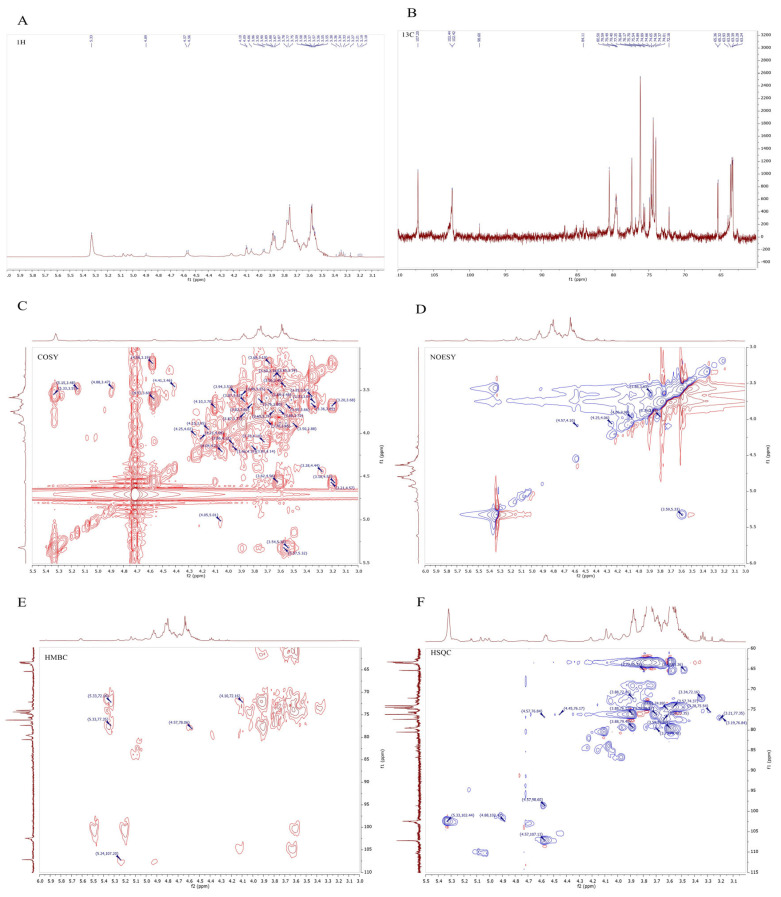
^1^H spectrum (**A**), ^13^C spectrum (**B**), ^1^H–^1^H COSY spectrum (**C**), NOESY spectrum (**D**), HMBC spectrum (**E**), and HSQC spectrum (**F**).

As shown in Figure 4A, the chemical shifts of the hetero head hydrogen atoms in CDP-D1 are δ 4.57,4.58,4.47,4.88, and 5.33. Based on the ^13^C NMR (Figure 4B) and HSQC (Figure 4F) spectra, the hetero head signals in the sample are identified as 5.33/102.44,4.88/102.42,4.58/107.13,4.57/98.6, labeled as sugar residues A, B, C, and D, respectively. Combining the information from the sample’s bonded structure, the heteroaromatic signals, and literature reports, it is inferred that sugar residue A is (→4,6)-α-D-Glcp-(1→), residue B is (→4)α-D-Glcp-(1→), residue C is (→2)-β-D-Galp-(1→), residue D is (→4)-α-D-GalpA-(1→).

Sugar residue A: The isomerization signal δ 5.33/102.44 ppm (H1/C1) indicates that residue B is likely an α-configured glucose residue. In the COSY spectrum (Figure 4C,D), the cross peaks δ 5.33/3.55 ppm identified residue B’s H2 (3.55 ppm), δ 3.55/3.69 ppm identified H3 (3.69 ppm), δ 3.69/3.89 ppm identified H4 (3.89 ppm), δ 3.89/3.49 ppm identified H5 (3.49 ppm), and δ 3.49/3.59 ppm identified H6 (3.59 ppm). These cross peaks are confirmed as chemical shifts of hydrogen atoms on the sugar ring. The HSQC signal assignments to the chemical shifts of carbon atoms on the sugar ring indicate residue B’s C1 shows δ 102.44 ppm, C2 δ 74.37 ppm, C3 δ 7.17 ppm, C4 δ 79.40 ppm, C5 δ 74.9 ppm, and C6 δ 78.06 ppm. Notably, the low-field shifts of C1, C4, and C6 suggest substitution at positions O-1, O-4, and O-6 of the sugar ring. Based on methylenation analysis and the literature, residue A is inferred to be (→4,6)-α-D-Glcp-(1→.

Sugar residue B: The isomerization signal δ4.88/102.42 ppm (H1/C1) indicates that residue B may be an α-configured glucose residue. In the COSY spectrum, the H2 of residue B (3.47 ppm) was identified from the cross peak δ4.88/3.47 ppm, the H3 (4.21 ppm) from the cross peak δ3.47/4.21 ppm, and the H4 (3.28 ppm) from the cross peak δ4.21/3.28 ppm, confirming their assignment to the chemical shifts of hydrogen atoms on the sugar ring. Subsequent HSQC signal analysis assigned the chemical shifts of C atoms on the sugar ring: C1 of residue B shows δ102.42 ppm, C2 δ65.36 ppm, C3 δ76.47 ppm, and C4 δ75.54 ppm. The low-field shifts of C1 and C4 suggest substitution at the O-1 and O-4 positions of the sugar ring. Combining methylation analysis results with literature reports, it is inferred that sugar residue B may be →4) α-D-Glcp-(1→.

Sugar residue C: The isomerization signal δ 4.58/107.13 ppm (H1/C1) for residue C indicates that it may be β-D-Galp. In the COSY spectrum, the cross peaks δ 4.58/4.10 ppm identified the hydrogen at position C (H2, 4.10 ppm), while the cross peaks δ 4.10/3.68 ppm identified the hydrogen at position C (H3, 3.68 ppm). The cross peaks δ 3.68/3.85 ppm identified the hydrogen at position B (H4, 3.85 ppm), and the cross peaks δ 3.85/3.70 ppm identified the hydrogen at position B (H5, 3.70 ppm). The cross peaks δ 3.70/3.21 ppm identified the hydrogen at position B (H6, 3.21 ppm), confirming these as chemical shifts of hydrogen atoms on the sugar ring. The HSQC signal was assigned to the chemical shift of carbon C on the sugar ring. The chemical shifts of residue B’s C1, C2, C3, C4, C5, and C6 were δ 107.13 ppm, δ 77.35 ppm, δ 70.07 ppm, δ 76.83 ppm, δ 76.17 ppm, and δ 77.35 ppm, respectively. The low-field shifts of C1 and C2 indicate substitution at the O-1 and O-2 positions of the sugar ring. Based on methylenation analysis and the literature, the sugar residue C is inferred to be →2)-β-D-Galp-(1→.

Sugar residue D: The isomerization signal δ4.57/98.6 ppm (H1/C1) indicates that residue D may be an α-configured galacturonic acid residue. COSY data suggest the H2 of residue D (3.60 ppm). Cross peaks δ3.60/3.34 ppm identified the H3 of residue A (3.34 ppm), while δ3.34/3.63 ppm confirmed the H4 of residue D (3.63 ppm). Cross peaks δ3.63/4.16 ppm further identified the H5 of residue D (4.16 ppm). HSQC signals assigned the chemical shifts of C atoms on the sugar ring: C1 of residue D shows δ98.6 ppm, C2 δ79.59 ppm, C3 δ77.38 ppm, C4 δ72.16 ppm, and C5 δ76.84 ppm. Due to weak signals in the NMR spectrum, complete assignment of all chemical shifts was not possible. Notably, the low-field shifts of C1 and C4 suggest substitution at O-1 and O-4 positions of the sugar ring. Combined with methylenation analysis results and literature reports, it is inferred that sugar residue D may be →4)-α-D-GalpA-(1→.

The sugar residue signals of CDP-D1 were assigned according to the 1D and 2D NMR spectra, as shown in Table 4.

Based on the chemical shifts of ^13^C and ^1^H of various sugar residues in CDP-D1, combined with NOESY (Figure 4D) and HMBC spectra (Figure 4E), the structural configuration and linkage patterns of the polysaccharide were analyzed. The HMBC correlation spectrum revealed the linkage mode between sugar residues: the remote coupling signal between the hetero-head hydrogen (H1, δH 5.33) of residue A and C2 of residue C (δC 77.35) indicates the formation of an A-(1→2)-C glycosidic bond. The cross-ring correlation peak between H1 of residue D (δH 4.57) and C6 of residue A (δC 72.16) demonstrates the branched linkage pattern of D-(1→6)-A. NOESY spatial correlation analysis showed a significant NOESY effect between H1 of residue A and H6 (δH 3.59), confirming the sugar ring’s C_1_ conformation. Additionally, the NOSEY signal between H1 of residue D and H2 of residue C (δH 4.10) further verified the spatial proximity of these linkage sites, thereby constructing the three-dimensional topological structure of the polysaccharide repeating unit.

The sugar residue signals of CDP-D1 were assigned according to the 1D (^1^H, ^13^C) and 2D NMR spectra (Figure 4). Complete chemical shift assignments for the four major residues are summarized in Table 4, with each assignment supported by cross-peaks from COSY, HSQC, HMBC, and NOESY experiments. Briefly, residue A (→4,6)-α-D-Glcp-(1→) was identified by its anomeric signal at δ 5.33/102.44 ppm and confirmed via HMBC correlation between H1 (5.33) and C2 of residue C (77.35 ppm). Residue B (→4)-α-D-Glcp-(1→) showed an anomeric signal at δ 4.88/102.42 ppm with low-field-shifted C4 (75.54 ppm) indicative of backbone 4-substitution. Residue C (→2)-β-D-Galp-(1→) exhibited a β-anomeric configuration (δ 4.58/107.13 ppm) and C2 low-field shift (77.35 ppm), consistent with 2-linked galactose. Residue D (→4)-α-D-GalpA-(1→), though partially obscured by weak signals, was assigned via HMBC correlation between H1 (4.57) and C6 of residue A (78.06 ppm), confirming its role as a side-chain appendage. Inter-residue linkages were further validated by NOESY spatial correlations, most notably between H1 of residue D and H2 of residue C (δ 4.10), establishing the three-dimensional topology of the repeating unit.

Based on the analysis of one-dimensional and two-dimensional NMR data combined with methylation results, it is inferred that CDP-D1 primarily consists of a backbone formed by interconnections of →4)-α-D-Glcp-(1→, →4,6)-α-D-Glcp-(1→, and a small amount of →4)-α-D-GalpA-(1→, →2)-β-D-Galp-(1→. The side chains are mainly composed of →4)-α-D-GalpA-(1→, →2)-β-D-Galp-(1→, which are attached to the O-6 positions of the →4,6)-α-D-Glcp-(1→ sugar residues. Therefore, the possible structure of CDP-D1 is hypothesized as follows:


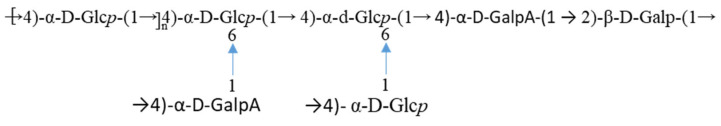


It is worth noting that the structural and biological insights obtained for CDP-D1 in this study differ significantly from those reported for the crude extracts or CDP-D2-N1 in our recent publication [18]. The high proportion of Glcp and galp, along with the specific glycosidic linkages identified via methylation and NMR, confer distinct rheological behaviors and a superior anti-inflammatory profile to CDP-D1 compared to the materials previously described. It should be emphasized that CDP-D1 is a polydisperse heteropolysaccharide, and the proposed model represents a consensus repeating unit that may not capture all microheterogeneity within the sample. Minor variant linkages or substitutions likely exist but fall below the detection threshold of current NMR methodologies.

### 3.10. Effect of CDP Polysaccharide on Cell Viability

NC (normal control group), LPS (lipopolysaccharide-induced inflammation model group), LPS+CDP (intervention group treated with CDP-D1 following LPS-induced inflammation), with cell viability in the NC group normalized to 100%, to investigate the protective effect of CDP on inflammatory cells and reflect the survival and functional status of cells after stimulation/treatment (higher viability indicates better cellular condition). Cell viability in the NC group was close to 100%, indicating that RAW264.7 cells maintain normal physiological function in the absence of external stimuli. Cell viability in the LPS group was significantly lower than that in the NC group, suggesting that the LPS-induced inflammatory response caused cellular damage or functional inhibition in RAW264.7 cells. Cell viability in the LPS+CDP group showed significant recovery relative to the LPS group and was nearly indistinguishable from the NC group, indicating that CDP-D1 treatment effectively reversed the LPS-induced decline in cell viability, restoring cellular function to baseline levels.

**Figure 5:**
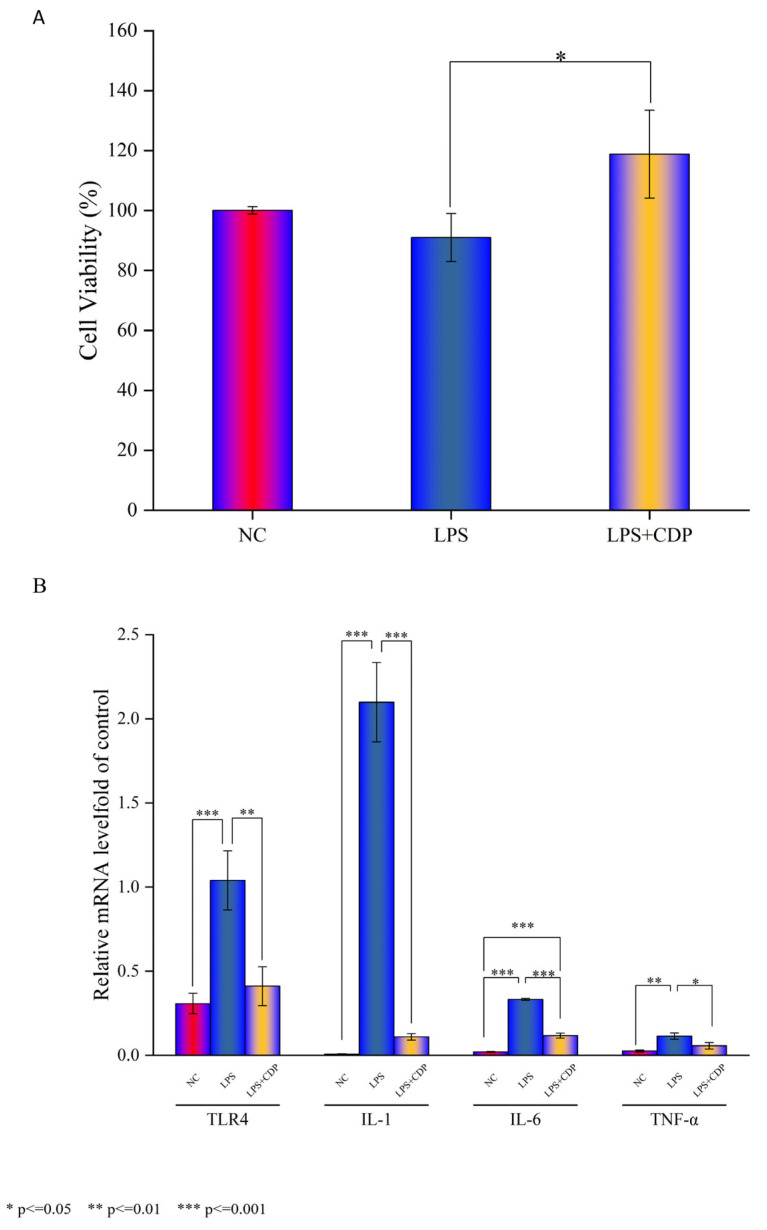
Anti-inflammatory effects of CDP-D1 on LPS-induced RAW264.7 cells. (**A**) Cell viability measured by CCK-8 assay. (**B**) Relative mRNA expression of TLR4, IL-1, IL-6, and TNF-α determined by qPCR.

As shown in Figure 5A, LPS successfully induced an inflammatory damage model in RAW264.7 cells (manifested as decreased cell viability); the CDP-D1 mediates a significant protective effect on LPS-induced inflammatory cells, restoring their function by enhancing cell viability to levels approaching the control group. RAW264.7 cells are macrophages; LPS stimulation triggers an excessive inflammatory response, ultimately leading to cellular damage. CDP-D1 mediates its effects by inhibiting pro-inflammatory signaling pathways such as NF-κB and MAPK, thereby reducing the release of pro-inflammatory cytokines like TNF-α and IL-6. Among these, TLR4 (Toll-like receptor 4), a central receptor in innate immunity and inflammation, functions as the “switch” for pro-inflammatory signaling pathways, and its high expression serves as a key marker of inflammation initiation. A comparison of TLR4 levels between the NC and LPS group shows that TLR4 mRNA levels in the LPS group were significantly higher than those in the NC group (*p* < 0.001), indicating that LPS stimulation activates the TLR4-mediated pro-inflammatory signaling pathway, successfully establishing an inflammatory model. Comparing the LPS group with the LPS+CDP group reveals that TLR4 mRNA levels in the LPS+CDP group were significantly lower than those in the LPS group (*p* < 0.01), indicating that CDP can block the initiation of pro-inflammatory signaling by downregulating TLR4 expression, thereby inhibiting the inflammatory cascade at its source.

A comparison of the NC group and the LPS group (Figure 5B) reveals that IL-1 mRNA levels in the LPS group were extremely significantly higher than those in the NC group (*p* < 0.001), reflecting that LPS stimulation can trigger a severe inflammatory response. Comparing the LPS group with the LPS+CDP group reveals that IL-1 mRNA levels in the LPS+CDP group were significantly lower than those in the LPS group (*p* < 0.001) and approached the levels observed in the NC group. This indicates that the polysaccharide CDP mediates a potent inhibitory effect on LPS-induced overexpression of IL-1 and can effectively mitigate the amplifying effects of the inflammatory cascade.

A comparison between the NC group and the LPS group reveals that IL-6 mRNA levels in the LPS group were significantly higher than those in the NC group (*p* < 0.001). Under LPS stimulation, IL-6 acts as a “key pro-inflammatory mediator,” participating in multiple pathological processes such as inflammatory amplification and immune imbalance. Comparison between the LPS group and the LPS+CDP group revealed that IL-6 mRNA levels in the LPS+CDP group were significantly lower than those in the LPS group (*p* < 0.001), indicating that the CDP can significantly reduce the pro-inflammatory toxicity of IL-6 by regulating its transcription, thereby restoring the inflammatory-immune balance.

A comparison between the NC group and the LPS group revealed that TNF-α mRNA levels in the LPS group (Figure 5B) were significantly higher than those in the NC group (*p* < 0.01), reflecting that, under LPS stimulation, TNF-α acts as an “initiator of the inflammatory cascade,” rapidly triggering downstream pro-inflammatory responses and tissue damage. Comparison between the LPS group and the LPS+CDP group revealed that TNF-α mRNA levels in the LPS+CDP group were significantly lower than those in the LPS group (*p* < 0.05), indicating that although the regulatory effect of the polysaccharide CDP on TNF-α was slightly less pronounced than that on IL-1 and IL-6, it could still significantly inhibit its overexpression and help block the progression of inflammation. LPS successfully induced a significant increase in the mRNA levels of TLR4 and downstream pro-inflammatory cytokines (IL-1, IL-6, TNF-α), establishing a typical inflammatory model. In contrast, the CDP mediates a clear anti-inflammatory effect by downregulating TLR4 expression (blocking the initiation of pro-inflammatory signaling) and inhibiting the transcriptional levels of IL-1, IL-6, and TNF-α (attenuating the “cascade amplification” effect of pro-inflammatory factors). Notably, CDP’s inhibitory effects on IL-1 and IL-6 are particularly pronounced, providing experimental evidence for the development of anti-inflammatory drugs or the evaluation of the bioactivity of natural products.

### 3.11. Partial Least Squares Discriminant Analysis

**Figure 6:**
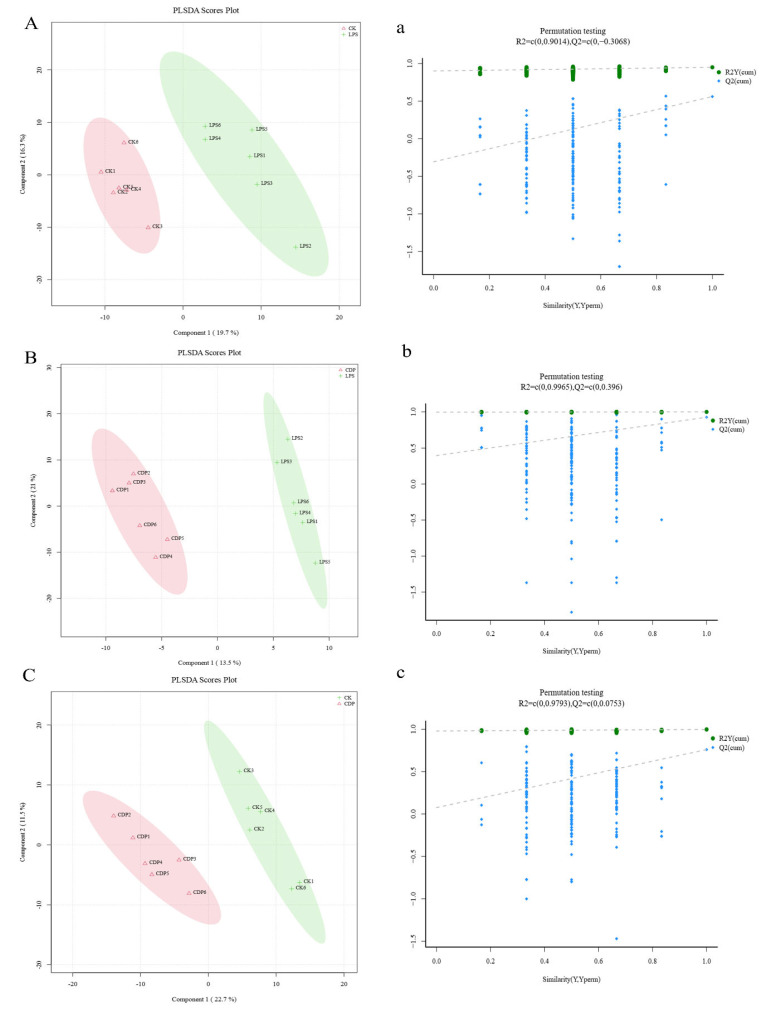
PLS-DA analysis of comparison groups and permutation test plot (**A**) PLSDA Score Plot of CK and LPS; (**a**) Permutation testing of CK and LPS; (**B**) PLSDA Score Plot of CDP and LPS; (**b**) Permutation testing of CDP and LPS; (**C**) PLSDA Score Plot of CK and CDP; (**c**) Permutation testing of CK and CDP.

As shown in Figure 6A, a comparative analysis between the CK and LPS groups demonstrates that the CK group comprises six samples and the LPS group comprises six samples (LPS1–LPS6). The *x*-axis represents Component 1 (19.7% variance explained) and the *y*-axis represents Component 2 (16.3% variance explained), cumulatively accounting for 35.6% of total variance. The spatial distributions of the two groups are well separated (CK group localized to the lower-left region, LPS group displaced toward the upper region) with no overlap. This suggests that LPS-induced inflammatory responses remodeled the metabolic profiles of RAW264.7 cells, thereby confirming the successful establishment of an inflammatory model.

As shown in Figure 6B, a comparison between the LPS and CDP groups reveals that the CDP group comprised six samples (CDP1–CDP6), and the LPS group comprised six samples (LPS1–LPS6). The *x*-axis represents Component 1 (13.5% variance explained) and the *y*-axis represents Component 2 (21% variance explained), cumulatively accounting for 34.5% of total variance. The data from the two groups were distinctly separated (CDP group positioned in the upper left, LPS group positioned in the lower right), indicating that CDP intervention modified the LPS-induced aberrant metabolic profile, though recovery was incomplete.

As shown in Figure 6C, a comparative analysis between the CK and CDP groups demonstrates that the principal component contributions were Component 1 (22.7% variance explained) on the horizontal axis and Component 2 (11.5% variance explained) on the vertical axis, cumulatively accounting for 34.2% of total variance (with Component 1 as the primary source of variance). Samples from the two groups formed distinct elliptical clusters (CK group localized to the lower left, CDP group scattered in the upper right), with limited overlap along the PC2 axis (a few CDP sample points approached the CK cluster boundary). This suggests that CDP treatment shifted the cellular metabolic phenotype closer to the normal state (CK group) but failed to achieve full normalization [39].

Permutation testing based on Figure 6a assessed PLS-DA model stability via R^2^Y (model explanatory power) and Q^2^Y (model predictive ability). Results demonstrated R^2^Y(cum) = 0.9965 (exceptionally high explanatory power) and Q^2^Y(cum) = 0.396 (moderate predictive ability). Green dots (R^2^Y) were concentrated around Y ≈ 1.0, while blue diamonds (Q^2^Y) were scattered around Y ≈ 0.0 and below. This suggests adequate model fit, though predictive ability requires evaluation in conjunction with other figures.

Figure 6b demonstrates that R^2^Y(cum) was concentrated at X > 0.2 and Y ≈ 1.0 (accounting for 0.9014 of variance), while Q^2^Y(cum) was dispersed between Y = −0.5 and 0.5 (with a predictive power of −0.3068). All Q^2^Y points were significantly below the random expectation diagonal (gray dashed line), suggesting the model’s predictive power was reliable (Q^2^Y was further reduced after permutation).

As shown in Figure 6c, R^2^Y(cum) was concentrated around X ≈ 0.3–0.4 and Y ≈ 1 (accounting for 0.9793 of variance), while Q^2^Y(cum) was distributed with an upward trend (predictive power 0.0753), thereby further validating model stability. These results demonstrate that the permutation test confirmed the PLS-DA model was free from overfitting, and intergroup difference analysis was reliable, rendering it suitable for subsequent metabolic mechanism studies [40].

Multivariate statistical analysis (PLS-DA + permutation test) demonstrates that the LPS vs. CK (successful inflammation model), LPS vs. CDP (effective polysaccharide intervention), and CDP vs. CK (partial recovery) groups were all clearly distinguishable through PLS-DA score plots. CDP exerted anti-inflammatory effects by reversing LPS-induced metabolic abnormalities, thereby providing a foundation for subsequent differential metabolite identification (e.g., LPS vs. CDP comparisons) and pathway enrichment analysis (e.g., energy metabolism and oxidative stress pathways).

### 3.12. Metabolite Classification Analysis

**Figure 7:**
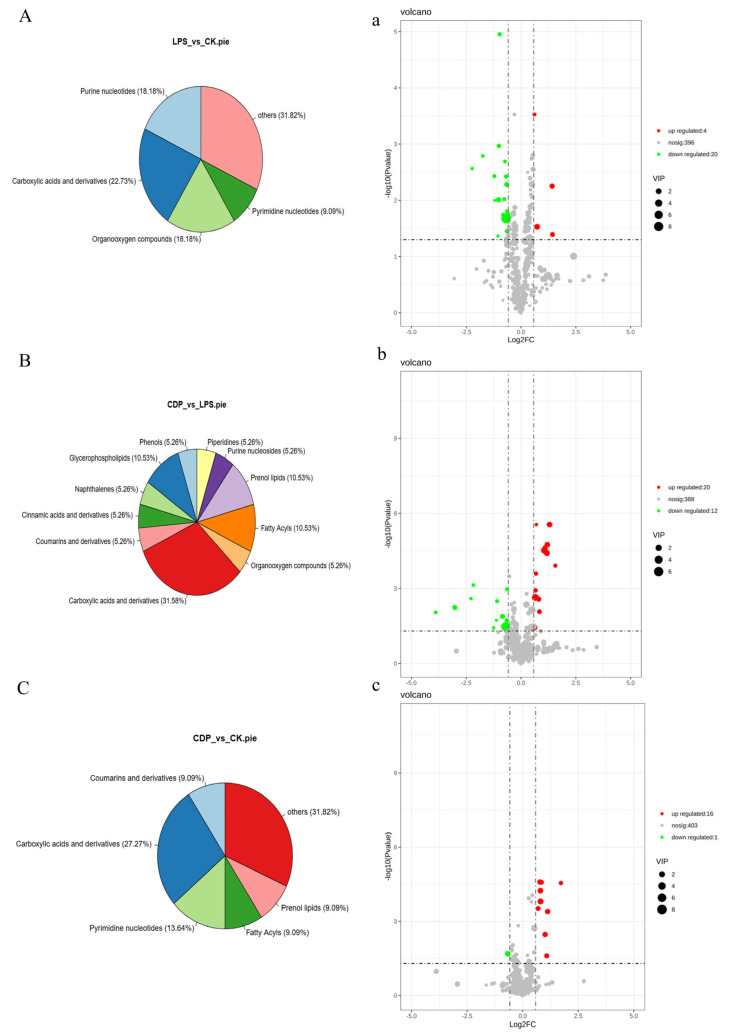
Metabolite classification pie chart and differential metabolite volcano plot. (**A**) pie chart of LPS and CK; (**a**) volcano plot of LPS and CK; (**B**) pie chart of CDP and LPS; (**b**) volcano plot of CDP and LPS; (**C**) P pie chart of CDP and CK; (**c**) volcano plot of CDP and CK.

As shown in Figure 7A (LPS_vs_CK.pie chart), purine and pyrimidine nucleotides collectively represented 36.36% (18.18% + 18.18%), significantly higher than other categories, reflecting heightened demand for nucleic acid synthesis and energy provision under inflammatory conditions. Carboxylic acids and derivatives (22.73%) and organooxygen compounds (18.18%) also exhibited elevated proportions, suggesting upregulated tricarboxylic acid (TCA) cycle activity and accumulation of oxidative stress products—consistent with the established inflammatory phenotype of LPS-stimulated macrophages.

In Figure 7B, the CDP vs. LPS. pie chart, the proportion of this category (carboxylic acids and derivatives) rose to 31.58% (higher than the 22.73% in the LPS group), while in the CDP vs. CK pie chart, it was 27.27% (higher than the 22.73% in the CK group but lower than the CDP vs. LPS group). These trends suggest that the polysaccharide (CDP) may alleviate LPS-induced energy depletion by upregulating the TCA cycle (boosting energy provision) while preventing excessive acidification. In the CDP vs. LPS. pie chart, glycerophospholipids, terpenoid lipids, and fatty acyls collectively represented 31.59% (significantly higher than that in the LPS group), indicating that CDP mediates anti-inflammatory effects via cell membrane repair and suppression of inflammatory lipid mediators.

The proportion of “others” in Figure 7C (CDP vs. CK pie chart) (31.82%) was identical to that in the LPS vs. CK pie chart, and the proportion of carboxylic acids and derivatives (27.27%) was closer to the 22.73% in the LPS vs. CK pie chart. This indicates that CDP treatment partially restored the metabolic profile to normal levels, though complete reversal may not have occurred due to constraints of intervention duration or dosage. Across all three group comparisons, this category consistently ranked as a core component (22.73% in LPS vs. CK, 31.58% in CDP vs. LPS, and 27.27% in CDP vs. CK), functioning as a key metabolic hub in the inflammatory and anti-inflammatory processes of RAW264.7 cells. These lipid categories were uniquely elevated in the CDP vs. LPS pie chart (with a combined proportion of 31.59%), indicating that CDP intervention specifically regulates lipid metabolism, potentially alleviating membrane damage through attenuation of oxidative modifications [41,42].

### 3.13. Analysis of Differential Metabolites

As shown in the volcano plot (Figure 7a–c), the three pairwise comparisons—CK (control group) vs. LPS (model group), LPS vs. CDP (polysaccharide treatment group), and CDP vs. CK—encompass a total of 420 metabolites.

As shown in Figure 7a, in the comparison between the LPS group and CK, 20 metabolites were significantly downregulated, accounting for 4.76% (20/420). Most *Log2FC* values were negative, indicating suppression of these metabolites upon LPS-induced inflammation. Four metabolites were significantly upregulated (0.95%, 4/420), with positive *Log2FC* values; however, their low abundance and low statistical significance (reflected by low *y*-axis values, i.e., low −log10(*p*)) suggest that LPS primarily suppresses most metabolites, activating only a minority. In total, 396 metabolites showed no significant differences (94.29%), reflecting stable expression of the majority under inflammatory conditions. Among the upregulated/downregulated points, some were of medium or large size (corresponding to VIP = 4/6/8), indicating that these 24 deferentially expressed metabolites (20 downregulated + 4 upregulated) are of high discriminatory importance for distinguishing the LPS group from CK and may serve as key inflammatory response biomarkers.

As shown in Figure 7b, in the LPS vs. CDP comparison, 16 metabolites were significantly upregulated (3.81%, 16/420), with *Log2FC* > 0 (expression restored/reactivated relative to LPS), suggesting CDP intervention reversed LPS-induced suppression. One metabolite was significantly downregulated (green dot; 0.24%, 1/420), likely representing an inflammation-related metabolite specifically inhibited by CDP. In total, 403 metabolites showed no significant differences (96.0%), indicating CDP mediates targeted effects with minimal off-target impact. Among the 16 upregulated metabolites, several exhibited medium-to-high VIP scores (VIP = 4/6), identifying them as core targets through which CDP reverses LPS-induced metabolic abnormalities.

As shown in Figure 7c, in the CDP vs. CK comparison, 20 metabolites were significantly upregulated (red dots; 4.76%, 20/420), possibly representing metabolites compensatory activated post-CDP treatment. Twelve metabolites were significantly downregulated (2.86%, 12/420), suggesting CDP may inhibit minor normal metabolic pathways. A total of 388 metabolites showed no significant differences (gray dots; 92.38%), indicating the CDP-treated metabolic profile approximated that of CK (majority of metabolites unchanged). Among upregulated/downregulated points, some had VIP = 6 (large dots), highlighting that these 32 differentially expressed metabolites (20 + 12) are of key importance for distinguishing the CDP group from CK.

The LPS group exhibited significantly more downregulated than upregulated metabolites versus CK (20 vs. 4), indicative of a dominant inflammation-induced metabolic suppression effect. The CDP group showed significantly more upregulated than downregulated metabolites versus LPS (16 vs. 1), suggesting polysaccharides exert effects primarily by activating and restoring metabolic function. In the CDP vs. CK comparison, metabolic profiles partially converged toward normal, yet exhibited “compensatory differences”: 92.38% of metabolites showed no significant differences, though 20 were upregulated and 12 downregulated. The LPS-induced cellular inflammation model was characterized by metabolic suppression (20 downregulated vs. 4 upregulated), with key differentially expressed metabolites (high VIP) serving as inflammatory markers. Following CDP intervention and reversal of dysregulation, 16 key metabolites (high VIP) were specifically activated, significantly reversing LPS-induced inhibitory effects (only 1 downregulated). Compared to CK, CDP approximated the normal metabolic profile (92.38% of metabolites unchanged), but 32 compensatory differentially expressed metabolites were identified, suggesting further optimization of treatment parameters is warranted.

### 3.14. Differential Metabolite Metepa Analysis

**Figure 8:**
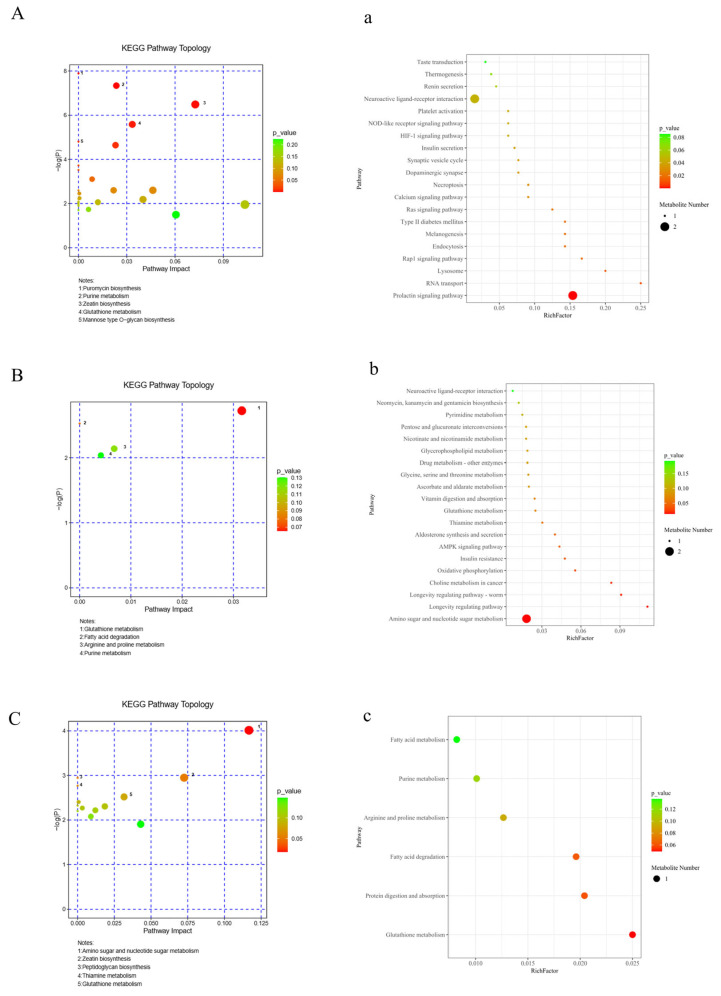
KEGG topology analysis bubble plot and KEGG pathway enrichment analysis bubble plot. (**A**) KEGG topology analysis bubble plot and KEGG of LPS and CK; (**a**) KEGG pathway enrichment analysis bubble plot of LPS and CK; (**B**) KEGG topology analysis bubble plot and KEGG of CDP and LPS; (**b**) KEGG pathway enrichment analysis bubble plot of CDP and LPS; (**C**) KEGG topology analysis bubble plot and KEGG of CDP and CK; (**c**) KEGG pathway enrichment analysis bubble plot of CDP and CK.

As shown in Figure 8A, the five data points comparing LPS and CK correspond to the following pathways: amino sugar and nucleotide sugar metabolism, zeatin biosynthesis, peptidoglycan biosynthesis, thiamine metabolism, and glutathione metabolism. All five points had *p*-values < 0.05, indicating that LPS-induced inflammation significantly altered these pathways. Notably, glutathione metabolism and purine metabolism (additional highlighted pathways) exhibited both high pathway impact and low *p*-values, with purine metabolism showing the most pronounced alteration, suggesting that LPS exerted the strongest effect on this pathway. The other four pathways—including glutathione metabolism—were also significantly altered, albeit to a lesser extent. These findings indicate that LPS stimulation perturbs multiple metabolic pathways, including glutathione-mediated antioxidant defenses and nucleotide sugar metabolism. Such perturbations are characteristic of the macrophage inflammatory phenotype but do not, in isolation, reveal the proximal molecular trigger of CDP-D1’s effects [39,40,41,42,43].

As shown in Figure 8B, in the CDP vs. LPS comparison group, the four dots correspond to the pathways: glutathione metabolism, fatty acid degradation, arginine and purine metabolism. The pathway impact for glutathione metabolism is approximately 0.03 (the highest among the pathways), indicating that CDP alleviates LPS-induced oxidative stress by enhancing antioxidant enzyme activity or supplementing glutathione precursors, thereby increasing the pathway’s impact value. Following CDP intervention, this pathway exhibited significant changes (with alleviation of LPS-induced oxidative stress). The pathway impact for purine metabolism is approximately 0.01, a notable decrease compared to its impact in the LPS vs. CK comparison, indicating that CDP effectively reversed the alterations in purine metabolism caused by LPS. In contrast, fatty acid degradation and arginine and proline metabolism showed lower significance, suggesting minimal effects of CDP on these pathways.

As shown in Figure 8C, the four pathways in the CDP vs. CK comparison are glutathione metabolism, fatty acid degradation, arginine and proline metabolism, and purine metabolism. Among these, glutathione metabolism and purine metabolism retained moderate pathway impact values and significant −log(*p*) values, indicating residual differences compared to the control group post-CDP treatment (primarily due to incomplete recovery of immune-related metabolism). Most pathways (e.g., fatty acid degradation) showed no significant differences, indicating that the CDP group and control group were comparable in these pathways, with the overall metabolic profile approaching normal levels.

MetPA analysis identified glutathione metabolism and purine metabolism as the pathways most responsive to CDP-D1 treatment. While these changes are consistent with attenuation of oxidative burden and partial restoration of metabolic homeostasis, they represent downstream correlates of CDP-D1 exposure rather than definitive evidence of a specific molecular target.

### 3.15. Differential Metabolite KEGG Pathway Enrichment Analysis

Figure 8a shows the comparison between the NC and LPS groups. The plot displays 5 data points corresponding to 5 metabolic pathways (not 20 as previously misstated): amino sugar and nucleotide sugar metabolism, zeatin biosynthesis, peptidoglycan biosynthesis, thiamine metabolism, and glutathione metabolism (per image annotations). The pathway impact ranges from 0 to 0.125, and −log(*p*) ranges from 0 to 4. Highly enriched pathways (Rich Factor > 0.15) were not observed in this plot; instead, pathways with moderate Rich Factor (0.03–0.05) and *p* < 0.05 (red/orange dots) indicated significant differences. For pathways with low enrichment (Rich Factor < 0.10, e.g., zeatin biosynthesis), dots were greenish (*p* > 0.05), suggesting no significant differences. Most pathways contained one differentially expressed metabolite (small dots), with a few containing two (large dots), indicating intergroup differences were primarily driven by single metabolites. This suggests that LPS-induced inflammation alters key pathways, and CDP reverses LPS-induced hyperactivation of glutathione metabolism and nucleoside sugar metabolism by inhibiting pro-inflammatory signals and enhancing antioxidant capacity [43].

Figure 8b shows the LPS vs. CDP comparison group, highlighting the relative enrichment of five core pathways (not six as previously misstated; per image: glutathione metabolism, fatty acid degradation, arginine and proline metabolism, purine metabolism, and an unlabeled pathway). Among these, glutathione metabolism had the highest Rich Factor indicating the most significant difference (LPS vs. CDP) and highest enrichment. This is because LPS induces oxidative stress, prompting cells to enhance antioxidant capacity via glutathione metabolism; however, the low *p*-value (*p* < 0.05, red dot) suggests this pathway is hyperactivated or imbalanced in the inflammatory model. CDP alleviates LPS-induced oxidative stress by enhancing antioxidant enzyme activity (e.g., SOD, GSH-Px) or supplementing glutathione precursors, thereby reducing the pathway’s enrichment. Amino sugar and nucleotide sugar metabolism was also significantly altered, reflecting LPS-stimulated immune cell activation requiring nucleotide sugars (e.g., UDP-glucose) for glycoprotein synthesis. This indicates LPS-stimulated macrophages undergo metabolic reprogramming characterized by increased demand for purine nucleotides to support biosynthetic and energetic demands during the inflammatory response [43]. This metabolic shift is a hallmark of activated myeloid cells and does not, by itself, implicate a specific upstream receptor or signaling cascade. Fatty acid metabolism had the lowest Rich Factor, indicating minimal differences and weak intergroup treatment effects.

Figure 8c shows the CDP vs. NC comparison group, displaying 4 data points corresponding to 4 metabolic pathways (not 20 as previously misstated): glutathione metabolism, fatty acid degradation, arginine and proline metabolism, and purine metabolism (per image annotations). The pathway impact ranges from 0.00 to 0.03, and −log(*p*) ranges from 0 to 2.5. Most pathways (3/4) showed no significant enrichment (*p* > 0.05, green dots), with small Rich Factor differences (0.005–0.03), indicating metabolic changes did not broadly affect these pathways. Only glutathione metabolism (pathway impact ≈ 0.03, -log(*p*) ≈ 2.5, *p* ≈ 0.07) and purine metabolism (pathway impact ≈ 0.01, −log(*p*) ≈ 2.1, *p* ≈ 0.11) retained moderate significance, suggesting residual differences post-treatment (e.g., incomplete immune metabolism recovery).

KEGG enrichment analysis systematically revealed metabolic pathway changes in RAW264.7 cells across the “normal–inflammation–polysaccharide treatment” continuum. LPS-stimulated macrophages exhibited significant enrichment of glutathione metabolism and amino sugar/nucleotide sugar metabolism, reflecting the oxidative stress and biosynthetic demands characteristic of the inflammatory phenotype. CDP-D1 treatment attenuated these LPS-induced perturbations, partially restoring the metabolic profile toward baseline levels. Notably, glutathione metabolism retained modest but detectable differences even after CDP-D1 treatment (Figure 8c), suggesting incomplete resolution of oxidative stress-related pathways within the experimental timeframe. While these metabolic shifts are consistent with the known biology of activated macrophages, they do not by themselves reveal whether CDP-D1 acts through a specific receptor-mediated cascade or via broader physicochemical interactions (e.g., antioxidant activity, membrane stabilization). The current data are therefore best interpreted as evidence that CDP-D1 modulates the inflammatory metabolic phenotype rather than as confirmation of a discrete molecular mechanism. CDP-D1 treatment attenuated the LPS-induced alterations in these pathways, consistent with partial restoration of metabolic homeostasis. However, the precise molecular interface between CDP-D1 and cellular signaling machinery remains to be elucidated through protein-level and pharmacological inhibition studies. Untargeted metabolomics identifies broad patterns of metabolic dysregulation and recovery, but cannot distinguish whether observed changes arise from direct interactions between CDP-D1 and cellular signaling machinery, indirect secondary effects of altered gene expression, or nonspecific physicochemical modulation. The identified pathways therefore serve as testable hypotheses for future targeted validation studies incorporating isotope tracing, enzymatic activity assays, or pharmacological inhibition experiments. Most pathways showed no significant enrichment (*p* > 0.05), with only a few key pathways exhibiting intergroup differences, demonstrating anti-inflammatory effects by regulating oxidative stress and immune metabolism [39,42,43].

## 4. Conclusions

CDP-D1 (95.5% purity, 33.1% yield) exhibits absorption peaks characteristic of polysaccharides; it is a heteropolysaccharide with an irregular amorphous structure, a porous and loose surface, and pseudoplastic flow behavior. The transition of CDP-D1 from a laboratory-scale extract to an industrial ingredient for food supplements and pharmaceuticals necessitates rigorous batch-to-batch consistency. Unlike synthetic drugs, the heterogeneity of botanical polysaccharides poses significant challenges. To address this, the consistency of CDP-D1 is ensured through a dual strategy involving process standardization and structural fingerprinting. This research systematically demonstrated the anti-inflammatory effects of CDP-D1 in LPS-induced inflammation in RAW264.7 macrophages and identified two key findings. First, CDP-D1 improved cell survival and function. Second, CDP-D1 inhibited pro-inflammatory signaling through a dual mechanism, including downregulation of TLR4 mRNA to block inflammation at its source and suppression of downstream cytokine transcription, significantly reducing the levels of IL-1, IL-6, and TNF-α to attenuate the cascade amplification effect. The study also elucidated the metabolic regulatory mechanism of CDP-D1 in LPS-induced inflammation in RAW264.7 cells. Metabolite classification and differential analysis revealed LPS-induced metabolic suppression and CDP-D1 mediated targeted restoration, underscoring CDP-D1’s role in reversing inflammatory dysregulation. Key pathways identified via Metepa and KEGG enrichment—glutathione metabolism and amino sugar/nucleotide sugar/purine metabolism—were central to CDP-D1’s anti-inflammatory effects. In summary, CDP-D1 attenuated LPS-induced upregulation of pro-inflammatory gene expression and partially normalized LPS-disrupted metabolic profiles in RAW264.7 macrophages. The observed modulation of glutathione and purine metabolism pathways suggests that CDP-D1 may alleviate inflammatory oxidative burden and support metabolic recovery. However, the current study is limited to transcriptional and metabolomic endpoints; direct evidence of interactions with specific signaling proteins awaits validation via Western blotting, immunofluorescence, or reporter assays. Future studies incorporating protein-level analyses and pharmacological antagonists will be essential to delineate the precise molecular mechanism underlying CDP-D1’s anti-inflammatory activity.

## Figures and Tables

**Table 2:** Fitting parameters of Cross model of membrane solution w of CDP-D1.

Sample Type	$\eta_{0}$/Pa.s	$\eta_{\infty }$/Pa.s	$\tau c_{r}$	n	R^2^
CDP-D1	13.9945	0.376402	3.29784	0.690664	0.993654

**Table 3:** Fitting parameters of Herschel–Bulkley model of CDP-D1 solution.

Sample Type	$\sigma_{y}$/Pa	$K_{HB}$	n	R^2^
CDP-D1	−0.554406	1.06758	0.854852	0.999590

**Table 4:** Chemical shifts of ^1^H and^13^C of sugar residues in CDP-D1.

Code	Glycosyl Residues	Chemical Shifts (ppm)					
		H1/C1	H2/C2	H3/C3	H4/C4	H5/C5	H6/C6
A	→4,6)-α-D-Glcp-(1→	5.33	3.55	3.69	3.89	3.49	3.59
		102.44	74.37	77.17	79.40	74.9	78.06
B	→4)α-D-Glcp-(1→	4.88	3.47	4.21	3.28	n.d	n.d
		102.42	65.36	76.47	75.54	n.d	n.d
C	→2)-β-D-Galp-(1→	4.58	4.10	3.68	3.85	3.70	3.21
		107.13	77.35	70.07	76.83	76.17	73.46
D	→4)-α-D-GalpA-(1	4.57	3.60	3.34	3.63	4.16	n.d
		98.6	79.59	77.38	72.16	76.84	n.d

n.d: an abbreviation for “not detected”, indicating no detection.

## Data Availability

The original contributions presented in this study are included in the article. Further inquiries can be directed to the corresponding author.
